# Differences in diversity and community composition of the shell microbiome of apparently healthy lobsters *Homarus americanus* across Atlantic Canada

**DOI:** 10.3389/fmicb.2024.1320812

**Published:** 2024-03-18

**Authors:** Svenja Koepper, K. Fraser Clark, J. T. McClure, Crawford W. Revie, Henrik Stryhn, Krishna K. Thakur

**Affiliations:** ^1^Department of Health Management, Atlantic Veterinary College, University of Prince Edward Island, Charlottetown, PE, Canada; ^2^Department of Animal Sciences and Aquaculture, Faculty of Agriculture, Dalhousie University, Truro, NS, Canada; ^3^Department of Computer and Information Sciences, University of Strathclyde, Glasgow, United Kingdom

**Keywords:** American lobster, aquatic microbiomes, 16S rRNA sequencing, alpha and beta diversity, UniFrac distance

## Abstract

Host-microbe dynamics are of increasing interest in marine research due to their role in host health and productivity. Changes in the shell microbiome of American lobsters have been associated with epizootic shell disease, a syndrome that is spreading northwards across the eastern U.S. and Canadian Atlantic coast. This study analyzed differences in alpha and beta diversity, as well as differentially abundant taxa, in the shell-associated bacterial community of apparently healthy lobsters from four lobster fishing areas (LFAs) in Atlantic Canada. Over 180 lobsters from New Brunswick, Nova Scotia and Prince Edward Island (PEI) were sampled during seven sampling events over four sampling months. The bacterial community was identified using novel PacBio long-read sequencing, while alpha and beta diversity parameters were analyzed using linear regression models and weighted UniFrac distances. The bacterial richness, diversity and evenness differed by sampling location, sampling month, and molt stage, but not by lobster sex or size, nor sampling depth. Similarly, based on LFA, sampling month, year and lobster molt stage, the shell microbiome differed in microbial community composition with up to 34 out of 162 taxa differing significantly in abundance between sampling groups. This large-scale microbial survey suggests that the shell microbial diversity of apparently healthy lobsters is influenced by spatial and temporal factors such as geographic location, as well as the length of time the carapace is exposed to the surrounding seawater.

## 1 Introduction

The microbiome is defined as the bacterial community colonizing a certain host-associated niche (Whipps et al., 1988). Decreasing sequencing costs and faster computational methods have facilitated the widespread use of microbiome research in recent years (Petersen and Osvatic, 2018). Studies on animal and human-associated bacterial communities are of particular interest as they are linked to host health and metabolism (Berg et al., 2015; Bikel et al., 2015; Cornejo-Granados et al., 2017) in such a way that some compare its functions to bodily organs (Baquero et al., 2012). Having a natural and healthy microbial community is thought to be beneficial for the host as it can protect against invading pathogenic bacteria, synthesize vitamins or help nutrient absorption (Ivanov et al., 2009; Venema, 2010; Bikel et al., 2015; Rajeev et al., 2021). Negative shifts in the microbial composition, also called microbial dysbiosis, have previously been associated with a range of diseases in animals and humans (Meres et al., 2012; Bhattacharjee and Lukiw, 2013; Mulle et al., 2013; Wynne et al., 2020). Defining the characteristics of a ‘healthy’ microbiome is not always obvious as there is high inter-individual variability even in clinically healthy patients. Bäckhed et al. (2012) described healthy microbiomes as having ecological stability to resist stress-induced community changes and being composed of taxa which provide functional benefits to the host. Many studies assume that microbiome health and resilience are indicated by an evenly distributed bacterial community that is high in richness and diversity (Sartor, 2008; Virgin and Todd, 2011; Bäckhed et al., 2012; Faith et al., 2013). However, these assumptions do not consider that some habitats, such as the vaginal microbiome, are dominated by only one genus (Lactobacillus) in their considered healthy state (Saraf et al., 2021).

Due to its commercial relevance, the American lobster (*Homarus americanus*) is one of the best-studied marine organisms (Goode et al., 2019). However, to this date, there have been few comprehensive studies on the shell microbiome of specimens in Atlantic Canada, although the shell microbial community has been linked to epizootic shell disease (ESD) (Meres et al., 2012; Smolowitz et al., 2014; Schaubeck et al., 2023). ESD is characterized by deep shell lesions on the carapace that can rapidly spread over the whole lobster body (Smolowitz et al., 2005). This decreases lobster survival and reproduction and has a negative impact on the lobster fishery, as affected animals have a lower market value (Castro et al., 2012). In the past, ESD outbreaks have impacted lobster populations and fisheries in the southern range of the species (Glenn and Pugh, 2006; Groner et al., 2018). Describing changes in the microbiome and potentially being able to link them to environmental or host factors could answer questions on host resilience and microbiome functions (Egan and Gardiner, 2016).

This study aimed to describe the shell microbial diversity and community composition of apparently healthy lobsters sampled from four locations in Atlantic Canada (New Brunswick, Nova Scotia and Prince Edward Island) using long-read PacBio sequencing for the 16S rRNA gene. The potential effects of sampling region and time were assessed, as well as lobster characteristics such as sex, size, or molt stage on the bacterial diversity, composition and bacterial species abundance. Furthermore, I wanted to determine any impact of different sampling sites (sampling from the boat vs. sampling directly from traps) on microbial diversity. Evaluating how environmental and host factors affect the lobster shell microbiome in the absence of shell disease is important to document the baseline microbial community of American lobster in Atlantic Canada.

## 2 Materials and methods

### 2.1 Sample collection

**Figure 1:**
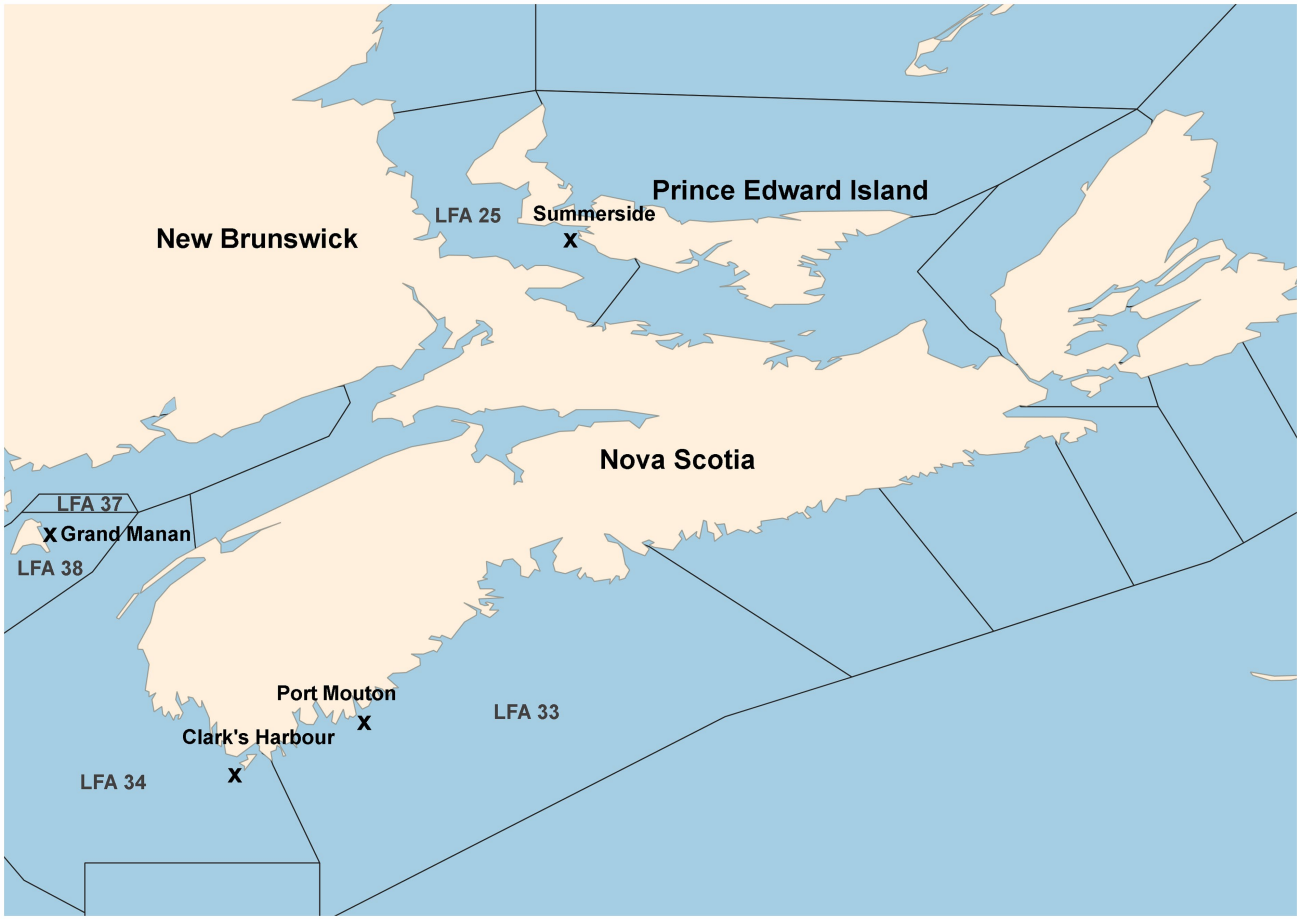
Map of sampling locations from commercial vessels or the wharf. Landing wharves are indicated by an “x.” Map after Koepper et al. (2023a).

A map of the sampling locations is provided in Figure 1. A detailed description of sample collection and processing has been given in Koepper et al. (2023a) which used the same microbiome data as in the present study. In short, carapace swabs of the lobster shell microbiome were collected in four LFAs within Atlantic Canada. In LFA 37, New Brunswick, one sampling event (May 2021); LFA 33, Nova Scotia, two sampling events (May and December 2021); LFA 34, Nova Scotia, two sampling events (May and December 2021); LFA 25, Prince Edward Island (PEI), two sampling events (September 2021 and October 2022). A direct comparison between samples taken from the boat and wharf was implemented in PEI (LFA 25 in October 2022). Swabs of the shell microbial samples were stored in 100% ethanol until further processing.

### 2.2 Bacterial 16S DNA extraction, sequencing, and bioinformatics

The methods and protocols used for bacterial 16S rRNA gene extraction, sequencing and bioinformatics correspond to the ones described in Koepper et al. (2023a). Briefly, DNA was extracted using the Qiagen Blood and Tissue kit according to the manufacturer’s instructions with a prior lysis step. The extracted DNA was shipped to the Integrated Microbiome Resource Lab (IMR) at Dalhousie University (Halifax, Canada) for full-length 16S rRNA gene sequencing (PacBio single-molecule real-time). All bioinformatics, such as dada2 denoising and filtering were completed in QIIME2 according to IMR protocols (Comeau et al., 2017). The produced amplicon sequence variants (ASVs) were taxonomically assigned using the trained Greengenes classifier v.138.99 (McDonald et al., 2011), after which all mitochondrial and eucaryotic ASVs were removed as well as ASVs that were not at least assigned to phylum level.

### 2.3 Statistical analysis

To identify differentially abundant bacterial taxa between sampling groups of interest (LFA, sampling month, sex, molt stage) the differential abundance method ANCOM II (version 2.1) (analysis of composition of microbes) was used previously described by Lin (2019). The ANCOM method accounts for the compositional nature of the microbiome data by using additive log ratios. This approach uses each taxon as a reference taxon successively (Mandal et al., 2015; Quinn et al., 2019; Nearing et al., 2022). Here, the non-rarefied, filtered ASV table (*N* = 189) containing read counts was used for inferring differentially abundant taxa with the R ANCOM-II workflow outlined by Lin (2019) and Nearing et al. (2022) including the R packages compositions (version 2.0–6) and nlme (version 3.1–162). Briefly, the feature table was pre-processed (function: feature_table_pre_process) to identify structural zeroes (taxon present in one sample but not in others) and outlier zeroes (not present in samples) (Kaul et al., 2017). Outlier zeroes were not considered during analysis, whereas structural zeroes were automatically assumed to be differentially abundant (Kaul et al., 2017; Nearing et al., 2022). After applying a pseudo count of 1, the dataset was log-transformed and statistical testing for all additive log ratios of each taxon was done using Wilcoxon rank-sum tests (Mandal et al., 2015; Nearing et al., 2022). The *p*-values were adjusted for multiple testing with the Benjamini-Hochberg method (Benjamini and Hochberg, 1995). Taxa were considered significantly differentially abundant if their number of rejections, denoted as *W_i_*, exceeded 70% of the maximum possible number of comparisons (Mandal et al., 2015; Lin and Das Peddada, 2020; Nearing et al., 2022). As described in Mandal et al. (2015), for this analysis it was assumed that the mean abundance of at most p-2 taxa are different between two populations. Accordingly, the differential abundances were tested by the following *p*(*p-*1)/2 hypotheses regarding the abundance of the *i*-th taxon relative to the *r*-th taxon for every *r* ≠ *i*:


$$H_{0ri}=E\left[\log\left(\frac{{\mu_{i}}^{\left(1\right)}}{{\mu_{r}}^{\left(1\right)}}\right)\right]=E\left[\log\left(\frac{{\mu_{i}}^{\left(2\right)}}{{\mu_{r}}^{\left(2\right)}}\right)\right]$$

To account for covariates, all other factors of interest were included in the ANCOM model when testing for differential abundance of taxa by groups. For example, to infer which taxa were significantly different by the four LFAs, sampling month, lobster sex and molt stage were adjusted for in the model.

To minimize bias due to differences in library sizes, each sample was rarified to 400 reads per sample for the subsequent diversity analyses. At this sequencing depth, 74,800 (5.82%) reads in 185 (97.89%) samples were retained and rarefaction curves indicated that microbial diversity (Shannon diversity) was fully captured (Supplementary Figure S1). Using this reduced dataset (*N* = 185), three alpha diversity indices – Chao1, Shannon diversity, and Pielou evenness – were calculated for each sample in QIIME2, using the rarefied and filtered ASV table containing read counts. Chao1 is an abundance-based index of species richness that uses non-parametric methods to account for taxa that are missing due to under-sampling using the observed species richness (Chao, 1984). The Shannon index is a commonly used diversity index, that estimates species richness and evenness by the proportion of the community represented by each taxon, i.e., taxon relative abundance (Shannon and Weaver, 1948). The Pielou evenness is indirectly based on Shannon diversity, i.e., relative abundance represents the ratio between observed values of the Shannon diversity and the Shannon value if all taxa had the same relative abundance (Pielou, 1966). To determine any effects of geographic and temporal as well as host factors on the shell microbial diversity, unconditional associations between the model outcomes (diversity indices) and each predictor (sampling events as a combination of LFA and sampling month, water depth, sex, size, molt stage, site) were checked in univariable linear regression models assuming a liberal *p*-value of 0.2 (Wald-test). Subsequently, multivariable linear regression models were fitted by a manual forward selection of significant predictors (Dohoo et al., 2009) for each of these indices, respectively. Two-way interactions between predictors were kept in the final model if they were below the significance threshold of *p* = 0.05. Pairwise comparisons of interest were assessed using the pwcompare command in Stata (v. 18, StatCorp. 2023^1^). Briefly, these comparisons investigated differences in the shell microbial diversity between sites (boat sampling vs. wharf sampling, only in LFA 25 in October 2022) and years (LFA 25), regions (LFA 34, 34 and 37 in May and December) and months (May and December in LFA 33, 34). The *p*-values were Bonferroni corrected for comparisons of interest, i.e., regional, seasonal, annual and site comparisons (*N* = 9). Normality and heteroscedasticity assumptions were checked by plotting the model residuals. To compare alpha diversity between sampling sites (boat vs. wharf sampling), linear regression models were fitted using data from LFA 25 (October 2022), where a direct site comparison was possible. Box plots of the diversity indices by LFA and months, molt stages and sites were plotted to visualize differences in microbial diversity between sampling groups. All regression modeling was done in Stata.

Differences in microbial composition between sampling groups were assessed by beta diversity analyses. The UniFrac method was adopted, in preference to other distance measures, such as Bray-Curtis and Jaccard, as this phylogenetic approach more effectively represents the distance between communities based on the lineages they contain (Lozupone and Knight, 2005; Lozupone et al., 2010). Weighted UniFrac considers taxonomic abundance by weighting the branch lengths of the phylogenetic tree by the relative abundance of the taxa (Lozupone et al., 2007). The weighted UniFrac distances were calculated from the rarefied and filtered ASV count table in QIIME2 based on the dissimilarity of the samples’ microbial composition. This was followed by Principal Coordinate Analyses (PCoA) to extract the dimensions accounting for the maximum distances by computing all eigenvectors and eigenvalues. Three-dimensional ordination plots were visualized using the emperor plot tool in QIIME2. Significant effects between LFAs, sampling months, sex and molt stage were assessed by a multifactorial PERMANOVA test (permutational multivariate analysis of variance) using the Adonis package in QIIME2 with 999 permutations (Oksanen et al., 2022).

## 3 Results

A total of 189 lobster samples were used for the analysis and detailed descriptive statistics such as number, size, sex, molt stage of sampled lobsters, sampling months, location, sampling site and water depths are shown in Koepper et al. (2023a) which used the same dataset (Supplementary Table S1, for rarefied data only). Briefly, a total of 1,277,587 reads (avg. 7,000 reads per sample) passed the bioinformatic filtering step such that the raw microbial dataset consisted of 5,173 features and 326 assigned ASVs. The rarefied library (normalized to 400 reads per sample) consisted of 3,588 features and 286 ASVs and four samples were dropped during rarefaction (insufficient sequencing depth) which resulted in 185 samples that were used for alpha and beta diversity analyses. At this sequencing depth, 74,800 (5.82%) reads in 185 (97.89%) samples were retained. A rarefaction curve is provided in Supplementary Figure S1. Overall, the shell microbiome of apparently healthy lobsters consisted for the most part of the bacterial classes *Gammaproteobacteria, Saprospiria, Verrucomicrobiae, Alphaproteobacteria, Flavobacteriia, Acidimicrobiia* and *Planctomycetia* (Koepper et al., 2023a).

### 3.1 Differential abundance analysis (ANCOM)

An ASV-focused differential abundance analysis was conducted to detect which taxa significantly differed between sampling groups. It showed that out of 162 taxa considered for differential abundance analysis, 69 taxa differed significantly between LFAs, 28 taxa between sampling months, 48 taxa between molt stages and only one taxon differed between sexes, when other covariates were adjusted for in multivariable analysis. The respective bacterial taxa (identified to genus and species level) are listed in Tables 1–3. For example, the *Alphaproteobacteria*, *Sulfitobacter mediterraneus* was only found in LFA 25, and there was a higher abundance in September 2021 than in October 2022 and with a more than 10 times higher relative abundance in postmolt lobsters. Similarly, *Candidatus* Endobugula (*Gammaproteobacteria*), *Glaciecola* (*Gammaproteobacteria*) and *Rubitralea* (*Verrucomicrobiae*) were more abundant in LFA 25 and in postmolt lobsters. On the other hand, *Hellea balneolensis* (*Alphaproteobacteria*), *Cocleimonas* (*Gammaproteobacteria*) and *Planctomyces* (*Planctomycetia*) were more abundant in LFA 33, 34, and 37 in May and December and in intermolt lobsters. An unclassified member of the *Acidimicrobiales* order was the only taxon that differed significantly in abundance between females, berried females, and males (data not shown).

**Table 1:** Taxa identified as significantly different between LFAs by ANCOM.

Class	Order	Family	Genus	Species	Relative abundance			
					LFA 25 *N* = 67	LFA 33 *N* = 51	LFA 34 *N* = 47	LFA 37 *N* = 24
*Cytophagia*	*Cytophagales*	*Flammeovirgaceae*	*Fulvivirga*	-	0.001	0.016	0.025	0.124
			*Gaetbulibacter*	*marinus*	0.465	0.017	0.006	0.000
			*Maribacter*	*-*	0.000	1.395	1.685	1.393
*Flavobacteriia*	*Flavobacteriales*	*Flavobacteriaceae*	*Polaribacter*	*-*	0.007	0.016	0.102	0.004
			*Ulvibacter*	*-*	0.168	0.067	0.038	0.003
			*Winogradskyella*	*-*	0.125	0.428	0.256	0.475
*Saprospiria*	*Saprospirales*	*Saprospiraceae*	*Portibacter*	*lacus*	0.007	0.173	0.155	0.275
*Planctomycetia*	*Planctomycetales*	*Planctomycetaceae*	*Planctomyces*	*-*	0.221	1.456	1.222	0.496
		*Hyphomonadaceae*	*Hellea*	*balneolensis*	0.012	0.380	0.338	0.111
			*Phaeobacter*	*-*	0.146	0.000	0.000	0.000
*Alphaproteobacteria*	*Rhodobacterales*	*Roseobacteraceae*	*Octadecabacter*	*antarcticus*	0.129	0.180	0.075	0.007
			*Roseovarius*	*aestuarii*	0.437	0.028	0.003	0.014
			*Sulfitobacter*	*mediterraneus*	0.185	0.000	0.000	0.000
*Deltaproteobacteria*	*Myxococcales*	*Nannocystaceae*	*Plesiocystis*	*-*	0.240	0.038	0.015	0.080
*Gammaproteobacteria*	*Alteromonadales*	*Alteromonadaceae*	*Cand.* Endobugula	*-*	3.710	1.596	1.243	1.333
			*Glaciecola*	*-*	0.100	0.001	0.001	<0.001
	*Beggiatoales*	*Leucotrichaceae*	*Cocleimonas*	*-*	4.994	8.867	11.161	7.581
			*MSBL3*	*-*	0.020	0.141	0.161	0.058
*Verrucomicrobiae*	*Verrucomicrobiales*	*Verrucomicrobiaceae*	*Rubritalea*	*-*	22.960	4.903	4.715	10.918
			*Verrucomicrobium*	*-*	0.010	0.090	0.115	0.082

**Table 2:** Taxa identified as significantly different between sampling months by ANCOM.

Class	Order	Family	Genus	Species	Relative abundance			
					May *N* = 71	Sep *N* = 24	Oct *N* = 43	Dec *N* = 51
*Flavobacteriia*	*Flavobacteriales*	*Flavobacteriaceae*	*Gaetbulibacter*	*marinus*	0.000	0.229	0.104	0.021
*Saprospiria*	*Saprospirales*	*Saprospiraceae*	*Lewinella*	-	0.002	0.109	0.028	0.017
			*Portibacter*	*lacus*	0.236	0.015	0.002	0.116
*Planctomycetia*	*Planctomycetales*	*Planctomycetaceae*	*Planctomyces*	-	1.200	0.305	0.173	1.144
*Alphaproteobacteria*	*Rhodobacterales*	*Hyphomonadaceae*	*Hellea*	*balneolensis*	0.406	0.012	0.012	0.178
			*Loktanella*	-	1.153	2.965	1.148	2.083
		*Roseobacteraceae*	*Octadecabacter*	*antarcticus*	0.017	0.100	0.145	0.228
			*Sulfitobacter*	*mediterraneus*	0.000	0.197	0.179	0.000
*Gammaproteobacteria*	*Alteromonadales*	*Alteromonadaceae*	*Cand. Endobugula*	-	0.739	5.089	2.940	2.340
*Verrucomicrobiae*	*Verrucomicrobiales*	*Verrucomicrobiaceae*	*MSBL3*	-	0.171	0.047	0.006	0.079
			*Verrucomicrobium*	-	0.115	0.028	0.000	0.074

**Table 3:** Taxa identified as significantly different between lobster molt stages by ANCOM.

Class	Order	Family	Genus	Species	Relative abundance	
					Inter *N* = 163	Post *N* = 26
*Flavobacteriia*	*Flavobacteriales*	*Cryomorphaceae*	*Crocinitomix*	-	0.274	0.479
			*Gaetbulibacter*	*marinus*	0.026	0.262
		*Flavobacteriaceae*	*Maribacter*	-	1.243	0.470
			*Ulvibacter*	-	0.047	0.343
*Saprospiria*	*Saprospirales*	*Saprospiraceae*	*Portibacter*	*lacus*	0.139	0.021
*Planctomycetia*	*Planctomycetales*	*Planctomycetaceae*	*Planctomyces*	-	0.922	0.311
*Alphaproteobacteria*	*Rhodobacterales*	*Hyphomonadaceae*	*Hellea*	*balneolensis*	0.234	0.026
		*Rhodobacteraceae*	*Loktanella*	-	1.429	2.907
			*Phaeobacter*	-	0.437	0.262
			*Roseovarius*	*aestuarii*	0.133	0.367
			*Sulfitobacter*	*mediterraneus*	0.029	0.297
	*Sphingomonadales*	*Erythrobacteraceae*	*Erythrobacter*	-	0.016	0.107
*Gammaproteobacteria*	*Marinicellales*	*Marinicellaceae*	*Marinicella*	-	0.275	0.623
	*Alteromonadales*	*Alteromonadaceae*	*Cand. Endobugula*	-	1.779	5.015
			*Glaciecola*	-	0.020	0.134
	*Beggiatoales*	*Leucotrichaceae*	*Cocleimonas*	-	8.563	3.756
			*Leucothrix*	-	0.745	0.487
*Verrucomicrobiae*	*Verrucomicrobiales*	*Verrucomicrobiaceae*	*Rubritalea*	-	10.116	23.964

### 3.2 Alpha diversity indices

**Figure 2:**
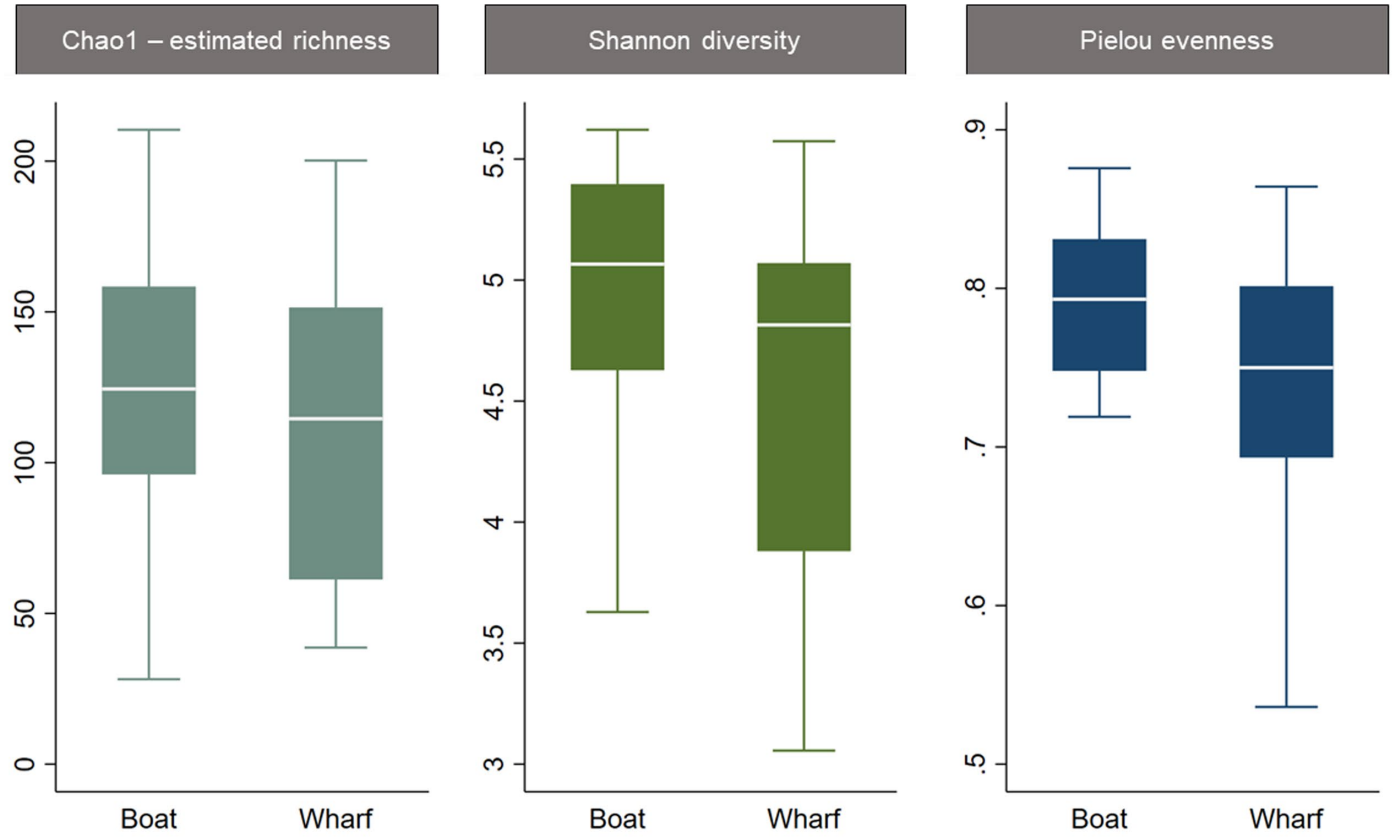
Boxplots of alpha diversity indices of the shell microbiome from lobsters by sampling site. Only samples from LFA 25 in October 2022 were included in this graph.

The three alpha diversity indices Chao1 (estimated richness), Shannon diversity and Pielou evenness were calculated, and linear regression modeling showed significant differences in microbial richness, diversity, and evenness by LFA and sampling month. Lobster size and sex, as well as water depth, were not significant in univariable regression for any alpha diversity indices and therefore excluded from the subsequent model-building process. The lobster molt stage had a significant effect on all microbial diversity indices in univariable analysis, with higher richness, diversity, and evenness in intermolt (lobsters with hardened shells between molting) compared to postmolt lobsters (lobsters with soft shells right after molting). However, the molt stage was not a significant predictor during forward multivariable model building and therefore not included in the final alpha diversity models. When comparing alpha diversity by sampling site (boat vs. wharf sampling) in LFA 25 (October 2022), samples from the wharf had a lower diversity (*p* = 0.026) and evenness (*p* = 0.021), but microbial richness did not differ between sampling sites (*p* = 0.289) (Figure 2). In the multivariable linear regression model with all sampling events, the sampling site was not a significant predictor and was excluded from the final model. No interaction terms were significant in the multivariable analyses (e.g., sex*size) and the final regression model consisted only of sampling events, e.g., the LFA and sampling months. Linear regression model outputs and parameters for each of the three alpha diversity indices as outcomes are summarized in Table 4.

**Table 4:** The factor variables and outputs of the linear regression models for the respective diversity indices.

		R^2^		0.503	
		Root MSE		64.36	
		F-statistic (7, 177)		27.59	
		Variables	Coefficient	SE	*p*-value
Chao1 – estimated richness	Sampling event	25 Oct (w)	−15.142	19.765	<0.0005
		25 Sep	33.881	18.998	
		33 Dec	84.349	17.904	
		33 May	194.289	18.781	
		34 Dec (w)	37.393	19.232	
		34 May	133.713	18.580	
		37 May (w)	28.957	18.580	
	Intercept		126.817	13.138	

	R^2^		0.570		
	Root MSE		0.55		
	F-statistic (7, 177)		35.9		
	Variables		Coefficient	SE	*p*-value
Shannon diversity	Sampling event	25 Oct (w)	−0.441	0.170	<0.0005
		25 Sep	0.218	0.164	
		33 Dec	1.086	0.154	
		33 May	1.658	0.162	
		34 Dec (w)	0.776	0.166	
		34 May	1.347	0.160	
		37 May (w)	0.618	0.160	
	Intercept		4.999	0.113	

	R^2^		0.507		
	Root MSE		0.05		
	F-statistic (7, 177)		28.0		
	Variables		Coefficient	SE	*p*-value
Pielou evenness	Sampling event	25 Oct (w)	−0.048	0.015	<0.0005
		25 Sep	−0.002	0.014	
		33 Dec	0.083	0.014	
		33 May	0.110	0.014	
		34 Dec (w)	0.076	0.015	
		34 May	0.088	0.014	
		37 May (w)	0.048	0.014	
	Intercept		0.791	0.010	

*N* = 185. MSE, mean squared error; SE, standard error.

**Figure 3:**
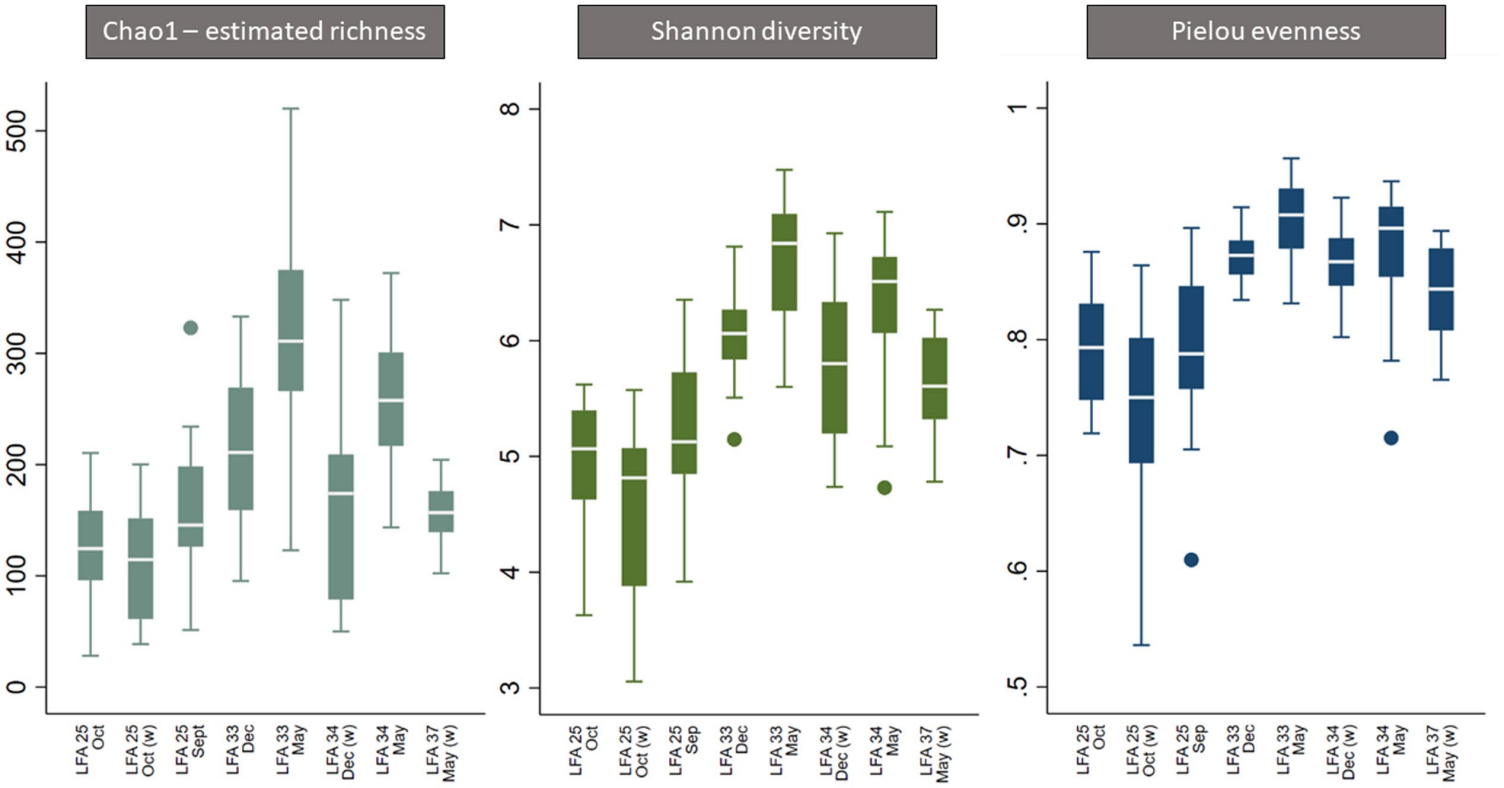
Boxplot of alpha diversity indices of the shell microbiome from lobsters sampled in four Atlantic Canada lobster fishing areas (LFA) by sampling month and sampling location [A suffix of “(w)” indicates that samples were taken from the wharf].

Overall, all three alpha diversity indices significantly differed by sampling events which is a combination of LFA and sampling month (Chao1: *p* = 0.000, Shannon: *p* = 0.000, Pielou: *p* = 0.000), and followed comparable patterns (Figure 3; Table 4). Microbial richness, diversity, and evenness were lowest in shell samples from LFA 25 with samples from the wharf (October 2022) having an even lower richness, diversity and evenness than samples taken from the boat in September 2021 and October 2022. Pairwise comparison (Bonferroni corrected) showed no significant difference in microbial richness, diversity, or evenness between samples taken in LFA 25 in September 2021 and a year later in October 2022, but highest in LFA 33 and 34 (May). When comparing the shell microbiome from boat and wharf samples (LFA 25, October) there was no significant difference in richness (*p* = 1.000) and diversity (*p* = 0.099), but microbial evenness differed between sampling sites (*p* = 0.018) in pairwise comparisons. Microbial richness, diversity, and evenness were highest in samples taken from LFA 33 and 34 in May, as compared to samples taken from the same LFAs in December. Pairwise comparison showed that in both LFA 33 and 34, microbial richness (LFA 33: *p* = <0.0005, LFA 34: *p* = <0.0005) and diversity (LFA 33: *p* = <0.0005, LFA 34: *p* = 0.009) differed significantly in May and December, but not evenness. However, when looking at the microbial composition in LFA 33 and 34 in the same months, in December neither richness, diversity nor evenness differed significantly between LFAs, but in May the estimated richness was significantly lower in LFA 34 than in LFA 33 (*p* = 0.018). The shell microbiome in LFA 37 (May) had similar microbial richness, diversity and evenness to samples collected in LFA 33 and 34 in December (all pairwise comparison *p* > 0.05), but not in May (LFA 33: *p* = <0.0005, LFA 34: *p* = <0.0005, for both richness and diversity). Results for all pairwise comparisons are shown in Table 5.

**Table 5:** Pairwise comparisons of shell microbial diversity between sampling events of interest.

	Comparison	Contrast	SE	95% CI		*p*-value*	CT
Chao1- Estimated richness	25 Oct (w) vs. 25 Oct	−15.16	19.83	−54.30	23.99	1.000	S
	25 Sep vs. 25 Oct	33.73	20.36	−6.45	73.90	0.891	A
	25 Sep vs. 25 Oct (w)	48.88	21.21	7.02	90.75	0.198	A
	33 May vs. 33 Dec	109.95	18.17	74.09	145.82	<0.0005	M
	34 May vs. 34 Dec (w)	96.36	19.35	58.16	134.55	<0.0005	M
	34 Dec vs. 33 Dec	−46.98	18.66	−83.80	−10.15	0.117	R
	34 May vs. 33 May	−60.58	18.83	−97.75	−23.41	0.018	R
	37 May (w) vs. 33 May	−165.33	18.83	−202.50	−128.16	<0.0005	R
	37 May (w) vs. 34 May	−104.76	18.63	−141.53	−67.98	<0.0005	R
Shannon diversity	25 Oct (w) vs. 25 Oct	−0.44	0.17	−0.77	−0.10	0.099	S
	25 Sep vs. 25 Oct	0.25	0.18	−0.09	0.60	1.000	A
	25 Sep vs. 25 Oct (w)	0.69	0.18	0.33	1.05	<0.0005	A
	33 May vs. 33 Dec	0.57	0.16	0.26	0.88	<0.0005	M
	34 May vs. 34 Dec (w)	0.56	0.17	0.23	0.89	0.009	M
	34 Dec vs. 33 Dec	−0.30	0.16	−0.62	0.01	0.531	R
	34 May vs. 33 May	−0.31	0.16	−0.63	0.01	0.513	R
	37 May (w) vs. 33 May	−1.04	0.16	−1.36	−0.72	<0.0005	R
	37 May (w) vs. 34 May	−0.73	0.16	−1.04	−0.41	<0.0005	R
Pielou evenness	25 Oct (w) vs. 25 Oct	−0.05	0.01	−0.08	−0.02	0.018	S
	25 Sep vs. 25 Oct	0.01	0.02	−0.02	0.04	1.000	A
	25 Sep vs. 25 Oct (w)	0.05	0.02	0.02	0.09	0.009	A
	33 May vs. 33 Dec	0.03	0.01	0.00	0.05	0.459	M
	34 May vs. 34 Dec (w)	0.01	0.01	−0.02	0.04	1.000	M
	34 Dec vs. 33 Dec	−0.01	0.01	−0.03	0.02	1.000	R
	34 May vs. 33 May	−0.02	0.01	−0.05	0.01	1.000	R
	37 May (w) vs. 33 May	−0.06	0.01	−0.09	−0.03	<0.0005	R
	37 May (w) vs. 34 May	−0.04	0.01	−0.07	−0.01	0.045	R

CT = comparison types (A = annual, M = monthly, R = regional, S = site), SE = standard error. (A suffix of “(w)” indicates that samples were taken from the wharf).

### 3.3 Beta diversity and ordination

**Figure 4:**
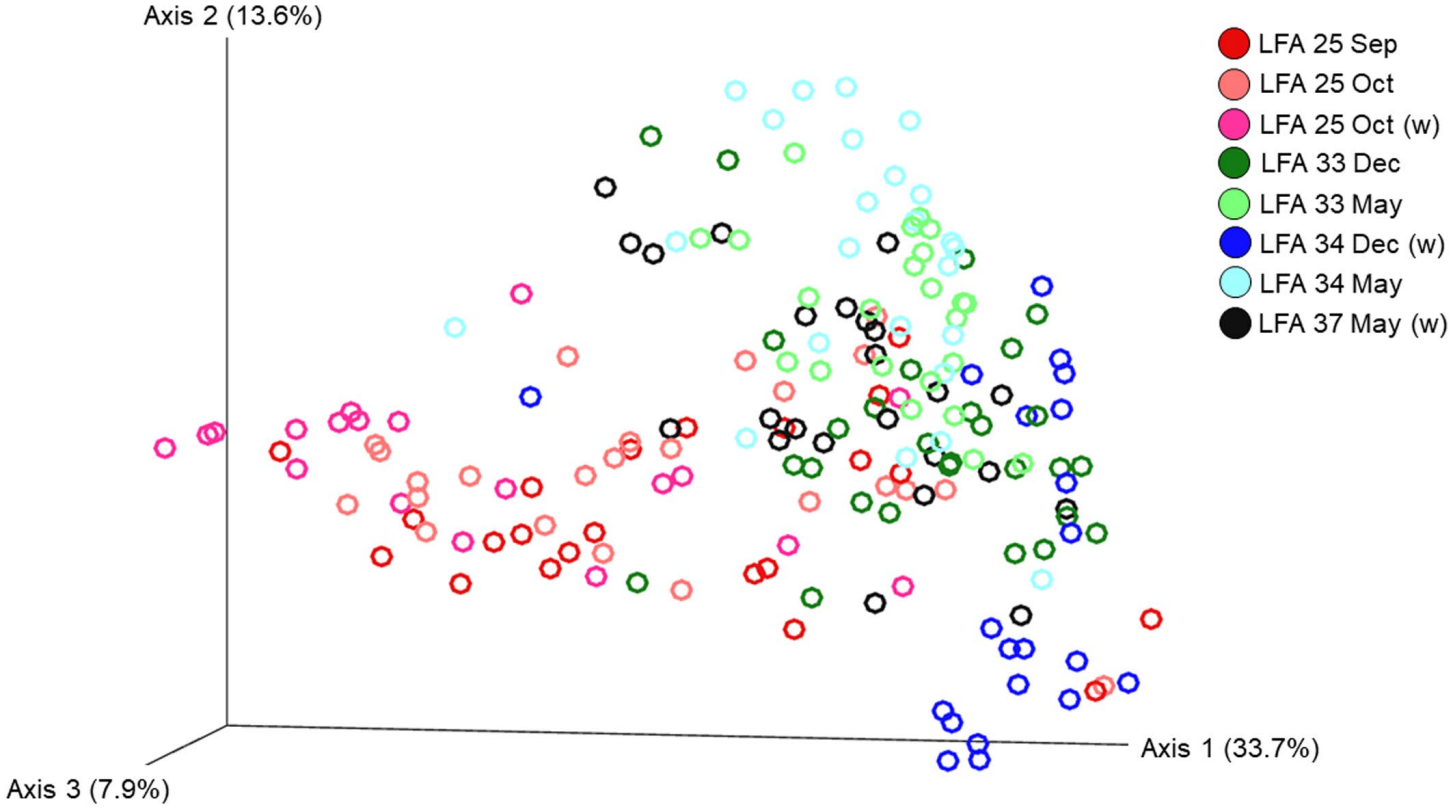
Ordination of the beta diversity of the shell microbial composition of lobsters sampled in four lobster fishing areas (LFA) in Atlantic Canada by LFA and sampling month using weighted UniFrac distances. Percentages represent the variation explained by the respective axis.

**Figure 5:**
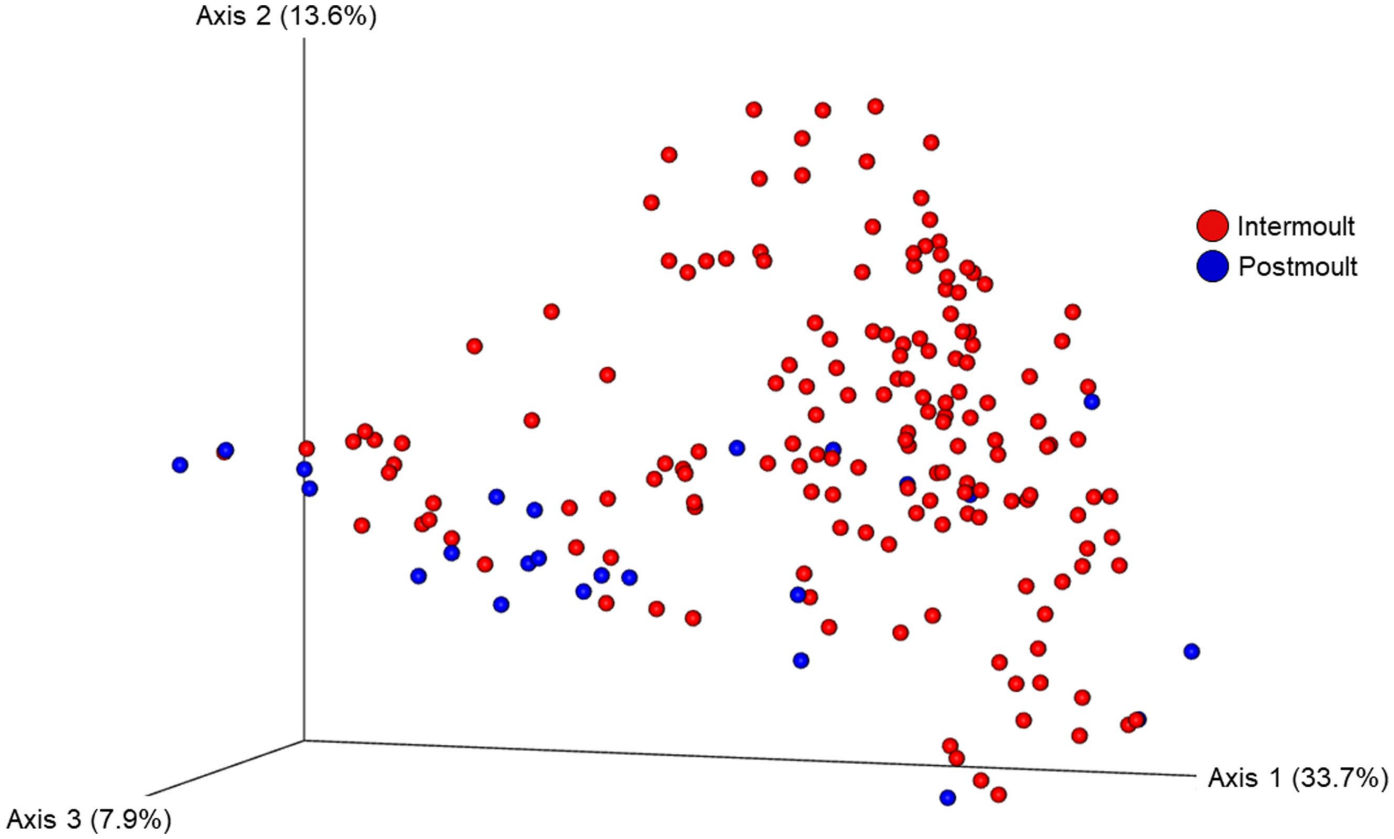
Ordination of the beta diversity of the shell microbial composition of lobsters sampled in four lobster fishing areas (LFA) in Atlantic Canada by molt stage using weighted UniFrac distances. Percentages represent the variation explained by the respective axis.

The ordination plot (Figure 4) shows the weighted UniFrac distances based on the similarity of the microbial community composition of the samples by LFA and sampling month. Samples with a similar microbial composition would have a closer distance to each other than samples that are dissimilar in their microbial composition. Samples from LFA 25 were distinctively clustered, and well separated from samples taken from LFA 33, 34, and 37. However, when only looking at samples taken from LFA 25, the microbial composition did not appear to differ by year (September 2021 vs. October 2022) or between boat and wharf samples (October 2022), as no distinct clustering was apparent. Samples from LFA 34 formed distinct clusters by season between shell microbiome samples from May and December. In LFA 33, this seasonal difference was not as pronounced. Overall, an overlap between LFA 33 and 34 samples could be observed. A multifactorial adonis PERMANOVA showed significant differences in microbial community composition by sampling event (a combination of LFA and month, *p* = 0.001, R^2^ = 0.55) and molt stage (*p* = 0.017, R^2^ = 0.55), but not by host sex (*p* = 0.313) (Table 6). The ordination plot by molt stage (postmolt and intermolt) does not show obvious clustering within the two sampling groups (Figure 5); though it should be noted that the number of postmolt samples was relatively limited.

**Table 6:** Multifactorial Adonis (PERMANOVA) of the beta diversity in the lobster shell microbiome based on weighted UniFrac distances.

	DF	SS	MS	PseudoF	R^2^	*p*-value*
Sampling event (LFA*Month)	7	3.823	0.546	14.036	0.354	0.001
Sex	1	0.044	0.044	1.120	0.004	0.313
Molt stage	1	0.109	0.109	2.802	0.010	0.017
Residuals	175	6.810	0.039		0.631	
Total	184	10.786			1	

DF, degrees of freedom; MS, mean sum of squares; SS, sum of squares. ^*^Significance values based on 999 permutations.

## 4 Discussion

This study presents a novel analysis of the shell microbial alpha and beta diversity of apparently healthy American lobster (*H. americanus*) in Atlantic Canada. These results are an important extension of the work published in Koepper et al. (2023a), which provided a novel description of the overall community composition. In this paper, we focus on microbial diversity patterns and differentially abundant taxa which may be an important indicator for host health. We found that microbial alpha diversity differs significantly by sampling region and months, but not annually within the same fishing area when sampled at a similar time of the year in fall (September vs. October) or by lobster sex or size. The molt stage also affected the microbial composition (beta diversity) of lobster shells but did not affect the alpha diversity after accounting for unbalanced sampling. Closing the knowledge gap of how, or if, shell microbial diversity impacts disease susceptibility. The presented results provide a promising baseline for future research on lobster shell disease and its management.

Alpha diversity indices for the shell microbial community were comparable to previously reported values for healthy American lobsters from Maine, US (Pielou evenness: 0.6–0.8) (Ishaq et al., 2023), from Long Island, US (Chao1: 211.5 ± 101.9, Shannon: 5.3 ± 0.9) (Schaubeck et al., 2023) and for spiny lobsters from New Zealand (Observed richness: 1308 ± 266, Shannon: 7.2 ± 1.4, Pielou evenness: 0.7 ± 0.1) (Zha et al., 2019). Interestingly, for rock and green crabs sampled from the Northumberland Strait (Malagash), near LFA 25 (Koepper et al., 2023b), a higher richness (Chao1: 650–1,000) and diversity (Shannon: 7.6–8.4), but a similar microbial evenness (Pielou: 0.83–0.86) than lobster shell samples from LFA 25 in the present study were observed. This study was the first to implement PacBio long-read sequencing of crustacean microbiomes. While Sadowsky et al. (2017) showed no significant difference in alpha diversity of human gut microbiomes between Illumina and PacBio platforms, it is unclear to which extent sequencing methods affect the comparability of aquatic microbiome surveys.

Furthermore, these results indicate that the shell microbiome does not substantially differ between lobster sex. Only one bacterial taxon had a significantly different abundance between male, female and berried female lobsters. Observing no significant differences in the alpha and beta diversity of the shell microbiome between lobster sex and size was somewhat surprising, as berried females and larger lobsters molt less often and have different preferred habitats (Cobb, 1976; Castro and Somers, 2012; Koepper et al., 2021). A longer time in their shell could imply a distinct microbial community as bacterial taxa, including pathogenic taxa, have more time to proliferate and colonize the carapace, but this was not observed here. While a lower number of berried females in this study could have masked microbial differences in the analyses, it is possible that microbial shifts are not observed in apparently healthy lobsters, but only closer to disease onset or in a transitionary state.

Significant differences in alpha and beta diversity and community composition of the shell microbiome were detected between different sampling months, sampling locations, and molt stages of the host. Seasonality has been shown to influence the skin microbiome of marine fishes, for example, Atlantic cod, killifish and Pacific chub mackerel sampled from the same location at different times of the year (Wilson et al., 2008; Larsen et al., 2015; Minich et al., 2020). Crustacean shell microbiomes have not been well studied in the absence of disease with a focus on environmental factors only, but microbial seasonality has been observed in wild swimming crabs (whole crab including carapace) and in the gut of copepods (Kim et al., 2017; Shoemaker and Moisander, 2017). A recent tank-based study on healthy and shell-diseased American lobster recorded lower microbial diversity (Shannon) in the winter compared to summer in healthy animals (Ishaq et al., 2023). Ward et al. (2017) showed that marine bacteria in the water column undergo seasonal shifts during the year and that these annual patterns are repeated in consecutive years. These findings are comparable to what was observed in this study where shell microbial diversity, richness and evenness were not significantly different between lobsters sampled in LFA 25 in 2021 and 2022 at the same time of year, but a seasonal difference was observed in lobsters sampled in May and December in LFA 34 and LFA 33. Similar to Ishaq et al. (2023), microbial diversity was lower in December compared to May. Water temperature has been proposed as a key driver of these seasonal fluctuations (Ward et al., 2017; Minich et al., 2018). It has the potential to directly impact the kinetics and physiology of bacteria and higher or lower temperatures can put selection pressure on the microbiome causing shifts in the community composition or altering richness, diversity and/or evenness (Beveridge et al., 2010; Ward et al., 2017).

These data suggested that geographic location also determined the shell microbial community structure, which was also supported by the highest number of differential abundant taxa in the ANCOM analysis by LFA. While comparable data for American lobsters are scarce, a similar study showed that the shell microbiome of rock crabs in Atlantic Canada had significant differences in microbial community structure based on the sample location (Koepper et al., 2023b). Sampling location combines several environmental factors such as water temperature, bottom type, ocean currents, salinity, pH and nutrient availability (not measured during the present study). It could be assumed that nutrient-rich regions tend to support more diverse microbial populations whereas low salinity (e.g., brackish conditions) may lead to a decreased microbial diversity due to higher selection pressure on marine microbes. Bottom temperatures in the Northumberland Strait (LFA 25) can reach up to 18°C and are warmer than in the other sampled LFAs in this study. Additionally, LFA 25 is less exposed to large ocean currents; for example, the cold and nutrient-rich Labrador Current passes by close to LFA 33 and 34 and ocean currents are proposed to shape aquatic host-associated microbiomes (Bundy et al., 2014; Debertin et al., 2018; Sousa et al., 2021). These factors may contribute to lower microbial diversity in LFA 25 compared to the other sampling regions. However, it is important to note that except for water depth, which did not have a significant effect on microbial diversity, no other environmental parameters such as bottom temperature or salinity were available for this study. Collection of these data in future marine microbiome studies could help to better understand microbial dynamics.

The lobster molt stage was the only host factor that influenced microbial beta diversity. While molt stage was not a significant factor after addressing confounding for alpha diversity (more postmolt lobster were sampled in LFA 25 (Koepper et al., 2023a)), the multifactorial weighted UniFrac analysis (PERMANOVA) indicated that the shell microbiome of inter- and postmolt lobsters differ in community composition. In Ishaq et al. (2023), where the lobster shell microbiome was monitored in a tank-based system over time, the molt stage also significantly affected the bacterial dissimilarity of the shell microbial communities. Here, different community compositions in intermolt lobsters could result from the longer time the shell was exposed to the surrounding seawater and its colonizing bacteria. In postmolt lobsters with “fresher” shells, fewer early bacterial colonizers potentially dominate the microbial community before more species can settle on the carapace and an equilibrium state is reached (Bäckhed et al., 2012).

Also, of interest was to compare the microbial sampling sites that were implemented in this study. Due to logistics and weather, some sampling events had to be conducted on the wharf. The results show that, while richness was stable, shell microbiome samples from the wharf had lower diversity and bacterial taxa were less evenly distributed. This somewhat contrasted the hypothesis that more handling on the boat would have increased microbial richness (e.g., contamination from gloves that also handle bait). Luo et al. (2022) showed that drying led to a decreased microbial diversity in river biofilms. Similar processes could be the reason for what was observed here on lobster shells sampled at the wharf, and it is important to acknowledge potential biases that can be introduced to microbiome datasets by altered sampling methods. However, the risk of contamination was minimized by rinsing the carapace surface with 0.2 μm filtered molecular biology grade water prior to swabbing to remove any incidental contamination from the native microbial biofilm.

Unfortunately, seawater control samples which were taken during sampling events, could not be analyzed, due to low bacterial DNA yield that prevented subsequent sequencing. Assuming that shell microbiome communities are shaped by the surrounding seawater and host factors which select beneficial bacteria (Koepper et al., 2023b), the analysis of water samples alongside aquatic host microbiomes should be encouraged for a more holistic understanding of microbial dynamics.

## 5 Conclusion

Using next-generation sequencing, it was demonstrated that microbial diversity and community composition on healthy lobster shells differed by sampling time and geographical region and to some extent, molt stage. Samples from postmolt lobsters, samples taken in winter months, and samples from LFA 25 in the Northumberland Strait were less rich, diverse, and even than samples from intermolt lobsters and animals that were sampled from the highly commercial lobster fishing areas in southwestern Nova Scotia and the Bay of Fundy in the month of May. It seems likely that a combination of environmental factors such as temperature, salinity, and habitat, as well as the host’s molt cycle and shell age, ultimately shape the microbial assemblages in such a way, that differences in microbial diversity are detectable over seasonal and spatial scales in Atlantic Canada.

As baseline data, these findings also raise the question of what increased or decreased microbial diversity means in terms of host health. A decrease in microbial diversity and richness precedes ESD and affected lobsters harbor a drastically altered carapace microbiome (Feinmann et al., 2017). With our current knowledge of lobster shell microbiomes, it can only be speculated whether differences in bacterial diversity in ESD-free lobsters are an indicator of host health or if they merely reflect how microbes naturally interplay with local abiotic and biotic factors. Including more microbial surveys in fisheries data collection, similar to what is done in aquaculture facilities nowadays, will enhance our understanding of highly relevant host-environment-microbiome interactions (Rajeev et al., 2021).

## Data availability statement

Publicly available datasets were analyzed in this study. This data can be found at: http://www.ncbi.nlm.nih.gov/bioproject/1026623.

## Ethics statement

The manuscript presents research on animals that do not require ethical approval for their study.

## Author contributions

SK: Writing – review & editing, Writing – original draft, Visualization, Methodology, Investigation, Formal analysis, Data curation, Conceptualization. KC: Writing – review & editing, Validation, Supervision, Methodology, Investigation, Conceptualization. JM: Writing – review & editing, Validation, Supervision, Methodology, Investigation, Conceptualization. CR: Writing – review & editing, Validation, Supervision, Methodology, Investigation, Formal analysis, Conceptualization. HS: Writing – review & editing, Validation, Supervision, Methodology, Formal analysis, Conceptualization. KT: Writing – review & editing, Validation, Supervision, Resources, Project administration, Methodology, Investigation, Funding acquisition, Data curation, Conceptualization.

## Footnotes

^1^ https://stata.com
