# Supplementary material for: Differences in diversity and community composition of the shell microbiome of apparently healthy lobsters Homarus americanus across Atlantic Canada
Source: Front Microbiol. 2024 Mar 18;15:1320812. doi: 10.3389/fmicb.2024.1320812 (PMC10986177; doi:10.3389/fmicb.2024.1320812)
Supplement: Supplementary file 1 [file Data_Sheet_1.docx]

Supplementary Material

Differences in diversity and community composition of the shell microbiome of apparently healthy lobsters *Homarus americanus* across Atlantic Canada

# Supplementary Table

**Table S1.** Descriptive and summary statistics of sampled apparently healthy lobsters of the rarefied dataset (N = 185). Median size (carapace length, in mm) and median water depth (in m) with 25^th^ (Q1) and 75^th^ (Q3) percentiles. B = boat, W = wharf; I = intermoult, P = postmoult, M = male, F = female, BF = berried female. Ch = Clark’s Harbour, GM = Grand Manan, PM = Port Mouton, S = Summerside. ^*^samples lost during rarefaction.

| **Province** | **LFA** | **Port** | **Date** | **N** | **Site** | **Median Size (Q1, Q3)** | **Moult stage (I/P)** | **Median depth (Q1, Q3)** | **Sex (M/F/B)** |
| --- | --- | --- | --- | --- | --- | --- | --- | --- | --- |
| NB | 37 | GM | May 2022 | 24 | W | 98 (89.5, 104.5) | (24/0) | NA | (13/10/1) |
| NS | 33 | PM | Dec 2021 | 28 | B | 94.5 (88, 104.5) | (27/1) | 28.0 (25.6, 29.3) | (19/7/2) |
|  |  |  | May 2022 | 23 | B | 86 (81, 92) | (23/0) | 10.4 (8.6, 11.9) | (11/10/2) |
|  | 34 | CH | Dec 2021 | 21^*^ | W | 88 (84, 93) | (19/2) | NA | (15/6/0) |
|  |  |  | May 2022 | 24 | B | 81 (77.5, 93) | (24/0 | 17.2 (9.5, 23.2) | (17/7/0) |
| PEI | 25 | S | Sept 2021 | 22 | B | 74.5 (73, 81) | (9/13) | 14.3 (14.3, 17.4) | (9/13/0) |
|  |  |  | Oct 2022 | 24 | B | 86 (78.5, 90.5) | (20/4) | 19.7 (18.1, 20.4) | (14/8/2) |
|  |  |  | Oct 2022 | 19 | W | 89 (84, 90) | (15/4) | NA | (11/8/0) |


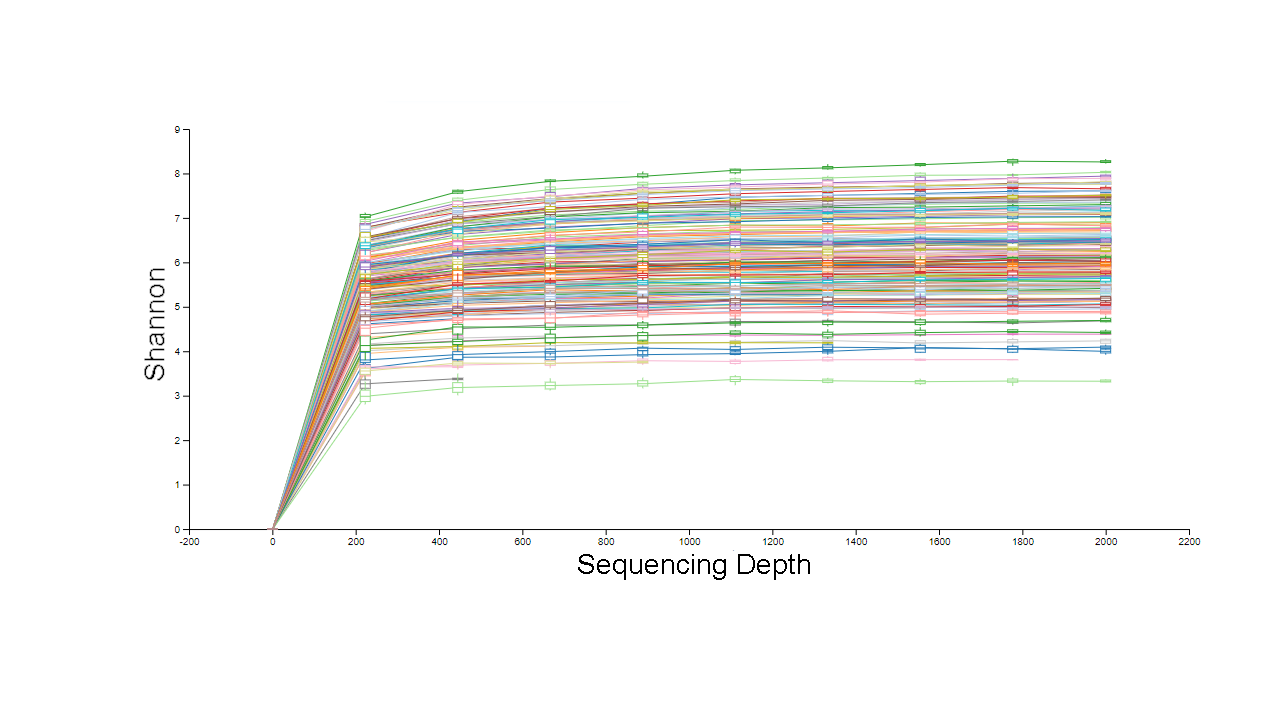


**Figure S1**. Rarefaction curves showing the Shannon diversity against the sequencing depth (reads per sample) where each line in the plots represents one sample. Plateauing lines indicate that the microbial diversity in the sample has been fully captured at the respective sequencing depth.
